# *Notes from the Field:* Mumps in Detention Facilities that House Detained Migrants — United States, September 2018–August 2019

**DOI:** 10.15585/mmwr.mm6834a4

**Published:** 2019-08-30

**Authors:** Jessica Leung, Diana Elson, Kelsey Sanders, Mona Marin, Greg Leos, Brandy Cloud, Rebecca J. McNall, Carole J. Hickman, Mariel Marlow

**Affiliations:** ^1^Division of Viral Diseases, National Center for Immunization and Respiratory Diseases, CDC; ^2^Public Health Safety and Preparedness Unit, DHS/ICE/ERO/ICE Health Service Corps; ^3^Emerging and Acute Infectious Disease Branch, Texas Department of State Health Services.

On October 12, 2018, five confirmed cases of mumps among migrants who had been transferred between two detention facilities were reported by the facilities to the Texas Department of State Health Services (TDSHS). By December 11, eight Texas detention facilities and six facilities in five other states had reported 67 mumps cases to U.S. Immigration and Customs Enforcement (ICE) Health Service Corps (IHSC) or local health departments. On December 12, TDSHS contacted CDC to discuss mumps control in detention facilities and facilitate communication with IHSC. During January 4–17, 2019, six more state health departments reported new cases in detention facilities, which prompted CDC and IHSC to launch a coordinated national outbreak response.

During September 1, 2018–August 22, 2019, a total of 898 confirmed and probable mumps cases (*1*) in adult migrants detained in 57 facilities (18% of 315 U.S. facilities that house ICE detainees*) were reported in 19 states (Figure); an additional 33 cases occurred among staff members. Private companies operated 34 facilities, 19 were county jails that house detained migrants, and four were ICE-operated. Forty-four percent (394) of cases were reported from facilities that house ICE detainees in Texas. Median patient age was 25 years (range = 17–67); 846 (94%) were male. Based on detainee custody status during their incubation period (12–25 days before symptom onset), most (758, 84%) patients were exposed while in custody of ICE or another U.S. agency^†^; 43 (5%) were exposed before apprehension; and the custody status at the time of exposure of 97 (11%) was unknown. Among those with data on complications, 79 (15%) of 527 male patients reported orchitis, and at least 13 patients were hospitalized. More than half (576, 64%) of cases were confirmed by quantitative reverse transcription–polymerase chain reaction testing or viral culture testing at CDC, state public health laboratories, Association of Public Health Laboratories–CDC Vaccine Preventable Disease Reference Centers, or commercial laboratories. Sequencing of isolates from 70 patients identified genotype G, the most common mumps genotype detected in the United States since 2006 (*2*). IHSC provided >25,000 doses of measles-mumps-rubella (MMR) vaccine in response to mumps in 56 facilities.

**FIGURE:**
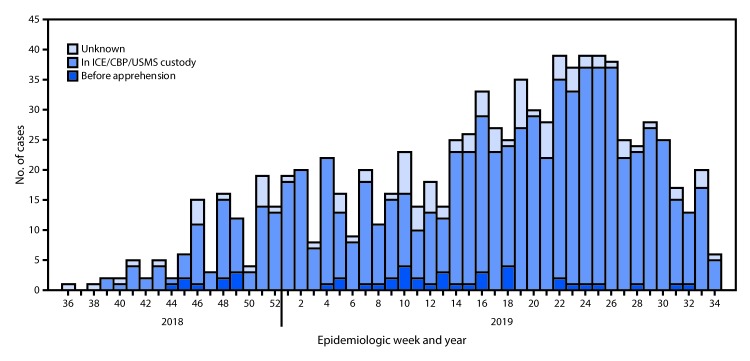
Mumps cases among U.S. Immigration and Customs Enforcement (ICE) detainees, by custody status* at time of exposure, by week of onset — United States, September 2018–August 2019 (N = 898)^†^ **Abbreviations:** CBP = U.S. Customs and Border Protection; USMS = U.S. Marshals Service. * Based on mumps incubation period of 12–25 days before symptom onset. ^†^ Data collected as of August 22, 2019.

Since 2015, approximately 150 mumps outbreaks and 16,000 cases have been reported in the United States, typically in close-contact settings such as universities, schools, and athletic events.^§^ This is the first report of mumps outbreaks in detention facilities.

MMR vaccination efforts differ among detention facilities; facilities should follow local or state health department recommendations for preventing and responding to mumps (*3*) and should report cases and follow disease control guidance from their health department. Detainees and staff members at increased risk for mumps should be offered MMR vaccine per existing recommendations for vaccination during outbreaks (*4**,**5*). MMR vaccine has not been shown to be effective at preventing disease in persons already infected with mumps; facilities should be aware that cases might occur among detainees exposed before vaccination.

Health departments, CDC, IHSC, and facility health administration can work together to develop appropriate control measures based on local epidemiology and the specific needs of each facility. Identifying and vaccinating close contacts of exposed or symptomatic persons with mumps in detention centers is challenging. IHSC can look up transfer history and facilitate vaccine procurement for detainees in ICE custody upon request from facility health services administrators. CDC is coordinating communication among state and local health departments, IHSC, and other federal partners to mobilize appropriate resources and is providing technical support for implementing appropriate disease control and prevention measures. Effective public health interventions require understanding of facility and custody operations, which often involve frequent transfers of detainees (between facilities and states) and multiple entities with authority for operations and detainee custody.

As of August 22, 2019, mumps outbreaks are ongoing in 15 facilities in seven states, and new introductions into detention facilities through detainees who are transferred or exposed before being taken into custody continue to occur.

## Footnotes

* Personal communication, Dr. Diana Elson, U.S. Immigration and Customs Enforcement. Facility count of 315 detention facilities on August 13, 2019, that housed ICE detainees with an average daily population >0 during Fiscal Year 2019. The number of facilities might change with time.

† U.S. Customs and Border Protection and U.S. Marshals Service.

§ https://www.cdc.gov/mumps/outbreaks.html.
